# Parental Pain Catastrophizing, Communication Ability, and Post-surgical Pain Outcomes Following Intrathecal Baclofen Implant Surgery for Patients With Cerebral Palsy

**DOI:** 10.3389/fpain.2021.809351

**Published:** 2022-02-22

**Authors:** Breanne J. Byiers, Caroline L. Roberts, Chantel C. Burkitt, Alyssa M. Merbler, Kenneth D. Craig, Frank J. Symons

**Affiliations:** ^1^Department of Educational Psychology, University of Minnesota, Minneapolis, MN, United States; ^2^Gillette Children's Specialty Healthcare, Saint Paul, MN, United States; ^3^Department of Psychology, University of British Columbia, Vancouver, BC, Canada

**Keywords:** catastrophizing, cerebral palsy, pain measurement, pain behaviors, parent child dyads, communication, observational pain assessment tools

## Abstract

There is strong evidence that psychosocial variables, including pain catastrophizing, influence parental and child ratings of pain, pain expression, and long-term outcomes among children with chronic pain. The role of these factors among children who have communication deficits due to cerebral palsy (CP) and other intellectual and developmental disabilities is currently unclear. In this study, parental pain catastrophizing was assessed before intrathecal baclofen (ITB) pump implantation for spasticity management in 40 children and adolescents with CP, aged 4 to 24 years. Pain was assessed before and after surgery with two methods: a parent-reported pain interference scale, and behavioral pain signs during a standardized range of motion exam. Linear mixed models with clinical/demographic factors and scores from the Pain Catastrophizing Scale for Parents (PCS-P), and child spoken language ability as predictors and the pain variables as the outcomes were implemented. On average, both pain outcomes improved after surgery. Only child spoken language ability predicted change in behavioral reactivity scores, with children with phrase speech showing an increase in reactivity at follow-up compared to pre-surgery levels, on average. A significant interaction between PCS-P scores and spoken language ability on change in pain interference scores over time showed that dyads with children with phrase speech whose parents reported high PCS-P scores reported the least improvement in pain interference at follow-up. Due to the preliminary nature of the study, future work is needed to investigate the parental behaviors that mediate the relationships between parental catastrophizing and pain outcomes in this population.

## Introduction

For children with complex communication needs resulting from intellectual and developmental disabilities (IDD) such as cerebral palsy (CP), parents play an essential role with regard to pain assessment and treatment. Cerebral palsy (CP) is the most common motor disability in children and musculoskeletal pain is very common in children and adolescents (1–3), and adults with CP (4). Because many individuals with severe CP cannot self-report their pain, particularly when cognitive impairment is also present, parents or other primary caregivers must interpret their child's behavior when they suspect that their child is experiencing pain to determine the severity and source of that pain, when to seek treatment, and what type of treatment to seek. It is well established that individuals with IDD frequently express pain in idiosyncratic ways (5), such as freezing or even laughing, so people who are unfamiliar may misinterpret these signs. As a result, clinicians often rely on caregivers to serve as proxy reporters for their child's pain, making their judgements a critical component of medical care.

Although parents and other primary caregivers are the most appropriate proxies for judging pain in children with IDD in most cases, many factors can influence both pain expression on the part of the child and judgements about the presence or severity of pain on the part of the caregiver. The Social Communication Model of Pain posits that the experience and expression of pain is the result of an integration of various biological, social, and psychological factors at the intrapersonal and interpersonal levels, involving not just the person experiencing pain, but also others present in the environment (6, 7). The model suggests that pain is not a simple biological construct, but a multifaceted dynamic process that is shaped by an individual's history and social environment.

For children and adolescents with (or without) severe disability, pain expression takes place within family microcultures. Repeated interactions and patterns of communication about pain over time have reciprocal influences on how pain is expressed and managed within the family system. Specific to children and adolescents with complex health care and communication needs who cannot reliably self-report, when considering proxy reports of pain, presumably the caregiver's own biological, affective, cognitive, and social factors influence both how the caregiver makes judgements about the presence and severity of pain, and the caregiver's behavioral responses to that pain. Relevant factors may include the caregiver's personal history of pain experiences, their sensitivity, biases, knowledge about pain and disability, and their relationship and perceived duties toward the child in pain (6). Such individual factors interact with dyadic parent-child variables and family level variables, according to family systems theory (8). Various moderators or mediators may be present, such as the child's age or developmental stage (9).

Among parents of children with chronic pain without IDD, parental behaviors, including modeling of pain behaviors and responses to child pain behaviors, have been shown to affect their child's pain expression, ratings of pain severity, and mental health and functional outcomes (10–13). In these studies, however, the children experiencing pain had no developmental or physical disabilities that affected their ability to communicate about their pain. It is therefore unclear how the psychosocial factors in the family environment affect pain expression and pain assessment among families of children with IDD, for whom conventional forms of communication are more difficult or absent.

Negative cognitive and emotional states with regard to pain have emerged as critical psychosocial variables in understanding individual differences in pain perception and experience. Although many different measures of pain cognitions exist, the self-report and parent-report versions of the Pain Catastrophizing Scale [PCS; (14, 15)] are among the most widely used. Catastrophizing is defined as “an exaggerated negative mental set brought to bear during actual or anticipated painful experience” [(16), p. 220]. People who are high catastrophizes judge pain stimuli as more threatening and express exaggerated pain reactions (17). Vervoort et al. (18) reported that catastrophic thinking moderated the relationship between parental presence/absence and facial expression of pain during an experimental pain paradigm among typically-developing children, suggesting potentially complex relationships between cognitive factors, the social context, and pain expression among typically-developing children.

Although it is typical and even adaptive in many cases for parents to worry about a child's pain, when worry becomes extreme it can become maladaptive, particularly in the context of chronic pain. Most studies have found that child pain catastrophizing is a stronger predictor of pain outcomes than parental pain catastrophizing among typically-developing children and adolescents with chronic pain. There is good evidence, however, that parent catastrophizing has an indirect influence on child outcomes primarily through its impact on child catastrophizing (19). In a triadic study of pain catastrophizing, Kraljevic et al. (20) found a significant positive correlation between the pain catastrophizing of fathers, mothers, and adult children. A systematic review found that parent catastrophizing was significantly related to increased child disability, depression, and parenting stress, yet weakly associated with child-reported pain intensity (21).

When the individual experiencing pain cannot advocate for themselves, the potential of parent catastrophizing to influence the parent's proxy report of their child's pain raises questions about the validity of these reports as accurate reflections of the child's pain experience. Parental psychosocial factors may also indirectly shape how children and adolescents express pain through the parent's behavioral responses to child pain behaviors. Therefore, the present study is a secondary data analysis exploring relationships between scores on the Pain Catastrophizing Scale for Parents in relation to pain outcomes in children and adolescents with CP with varying degrees of communication abilities. This was done in the context of a study primarily designed to evaluate the impact of surgical implantation of intrathecal baclofen (ITB) pumps on pain outcomes among children and adolescents with severe spasticity due to CP [see (22, 23)]. ITB implantation has been shown to reduce spasticity and parent-reported pain among individuals with CP (24, 25), but no studies have examined the influence of caregiver factors on pain outcomes in this population. We anticipated that the relationships between parental pain catastrophizing and the pain measures, including change over time, might differ by child communication ability. We hypothesized that parental pain catastrophizing would be positively correlated with parental pain interference ratings and behavioral expression of pain at a group level, and that both measures would show significant decreases following ITB implantation.

## Method

### Participants

Parent-child dyads in which the children had clinical diagnoses of CP and were scheduled for ITB implantation at a specialty pediatric hospital were eligible for participation in this prospective cohort study. The sample represents a clinical convenience sample formed through consecutive enrollment based on scheduled ITB pump implant surgery. A total of 63 dyads participated between October 2013 and March 2019. For the current analyses, dyads were excluded if: the Pain Catastrophizing Scale was missing because the caregiver did not speak English fluently enough to complete it (*n* = 2), or due to time constraints (*n* = 2), the adult who attended the surgery and completed the initial questionnaires was not a primary caregiver for the child (*n* = 4), the same caregiver did not complete the questionnaires at all visits (*n* = 3), or the dyad did not complete any follow-up assessments within 90–280 days (~3 to 9 months) following the implantation (*n* = 6). In addition, preliminary analyses suggested that patterns for both the parent-reported and direct observational measures differed between male and female caregivers. Because the sample of fathers who participated in the study was too small to provide stable estimates (*n* = 5), these dyads were excluded from the analyses, so all participants represent mother-child dyads. Finally, data from visits that occurred within 3 months of a major surgery or procedure, or during which parents reported acute pain, such as due to acute illness or injury, in the previous week were excluded to ensure that pain scores reflected primarily the influence of chronic pain. This resulted in the exclusion of one additional participant with no study follow-up study visits without reported acute pain. A final sample of 40 dyads contributed two valid data points for the pain scores. Of these participants, 32 completed at least one in-person follow-up assessment in the 3 to 9-month post-operative period. Observational data from two participants were not usable due to technical difficulties and/or challenges obtaining clear views of the participant's face during the standardized exam at one or both time periods, and one participant had acute pain at both follow-up visits during the time window. The final sample for the direct observation analyses was therefore 29. Demographic and clinical factors by communication status are reported in Table 1.

**Table 1:** Participant dyad demographics by analysis.

**Demographic and clinical variables**	**Behavioral reactivity and proxy report (*****N*** **=** **29)**			**Proxy report only (*****N*** **=** **40)**		
***Categorical variables***	***n***	***%***		***n***	***%***	
**Child sex**						
Male	16	55		24	60	
Female	13	45		16	40	
**GMFCS level**						
II or III	5	17		8	20	
IV	5	17		9	23	
V	19	66		23	58	
**Intellectual disability**						
None	2	7		3	8	
Mild/moderate	11	38		18	45	
Severe/Profound	16	55		19	48	
**Spoken language**						
No phrase speech	23	79		30	75	
Phrase speech	6	21		10	25	
Parent college degree	21	72		28	70	
Race = white, not Hispanic, Latinx	22	76		32	80	
***Continuous variables***	***Mean***	***SD***	***IQR***	***Mean***	***SD***	***IQR***
Child age (months)	132.83	50.29	99–155	131.73	53.65	96–153
PCS-P scores	37.45	13.11	27–49	38.13	12.00	31–47
**Behavioral react. scores**						
Before surgery	23.28	7.30	18–28	23.47	7.35	18–28
After surgery	19.93	7.71	16–25	18.71	7.74	16–25
**Pain interference scores**						
Before surgery	43.59	34.18	12–67	41.68	32.63	8.5–65
Follow-up	25.07	26.47	6–36	22.23	23.91	18–29

### Procedure

As part of a larger prospective intrathecal baclofen (ITB) outcomes project, parent-reported psychosocial assessments were completed before ITB implantation for spasticity management. Assessments regarding pain and comfort, including the standardized pain exam, were completed prior to surgery and again at ~3, 6, and 9 months post-surgery. Parents completed the initial measures on an iPad with the assistance of a researcher. Parents completed the follow-up measures on their own *via* an online REDCap survey. The direct observational assessment was completed in clinic areas while the participants were present prior to surgery and at follow-up standard of care appointments. The 3 to 9-month window for follow-up was selected for the current analyses because previous studies have shown that ITB results in decreases in pain within this period (23, 26) the initial 3-month visit was for complete post-surgical recovery, and in general, the time window minimized the risk of intervening surgeries or other health events that could interfere with the results. If participants had two time points with valid data within the selected 3 to 9-month window, the date closest to the 180 days post-surgery was selected for analysis. The average time to follow-up after surgical implant was 172 days (range = 95–251) for parent questionnaires and 175 days (range = 99–251) for the direct observational measure.

### Measures

#### Pain Interference

A modified version of the Pain Interference subscale of the Brief Pain Inventory (BPI) as described by Tyler et al. (27) was used to assess the degree to which parents perceived that ongoing pain interfered with daily living for their child. The scale includes 10 items, each rated on an 11-point scale (0 = pain did not interfere, 10 = pain completely interfered). The items include general activity, mood, mobility, work school or chores, relationships with other people, sleep, enjoyment of life, self-care, recreational activities, and social activities (27). A previous study examining the psychometric properties of this modified version of the BPI reported that scores were significantly correlated to other proxy-completed pain assessments, such as the Dalhousie Pain Inventory (28). In our sample, Cronbach's alpha of the BPI was 0.97 at Time 1 and 0.98 at Time 2.

#### Pain Catastrophizing Scale for Parents

The PCS-P (assessed at Time 1 only) is a 13-item scale characterizing thoughts and feelings that parents may experience when their child is in pain (14). Parents rate the frequency with which they experience thoughts and feelings on a 5-point scale (0 = “not at all”, 4 = “extremely”). In our sample, Cronbach's alpha of the PCS-P was 0.93.

#### Spoken Language Ability

Participants with CP were grouped into three groups according to their spoken language ability based on parent-reported verbal ability (phrased as a yes/no question), and observation of the child's spoken language during the study visits. Participants who were reported to not use spoken language were categorized as “none”, those who were reported to use spoken language were grouped into “some words” or “phrase speech” based on observations of the participants during study visits. Participants who did not speak during the visits, or who spoke in brief utterances (i.e., no more than two words) were categorized as using “some words”, and those who uttered at least one three-word utterance during the visit were categorized as having “phrase speech”.

#### Demographic Information

Additional demographic information, including degree of cognitive/intellectual impairment (i.e., no impairment, mild/moderate, or severe/profound), child's date of birth, parental sex and educational attainment, and race/ethnicity were collected *via* a parent survey.

#### Gross Motor Function Classification Scale

The GMFCS is designed to provide an objective classification of motor disability in children with CP, with an emphasis on sitting and walking. Function is divided into five levels, with children at Level I having the most independent motor function and Level V having the least (29). Because a large majority of the children in the current study were functioning at GMFCS levels IV or V, those in levels II and III were grouped together for analysis purposes.

#### Pain Examination Procedure

A standardized range-of-motion pain examination procedure (PEP) was completed at both time points. The PEP was designed to identify potential sources of pain or discomfort, such as spasticity or gastrointestinal pain. This exam has been used in previous studies of assessing pain- and discomfort- related nonverbal behavioral reactivity to experimental pain in IDD, including Rett syndrome, a neurodevelopmental disorder with associated motor impairment (30). The PEP involved the examiner slowly moving each joint of the arms and legs through its full range of motion in a standard sequence. The exam also included rotation of the head to each side, but due to the face being difficult to score during the head movements, reactivity was only scored for the arm and leg portions. The procedure was video recorded for later scoring by trained observational coders.

#### Direct Observational Scoring

Behavioral reactivity was scored for each limb of the PEP using a modified version of the Pain and Discomfort Scale (31–33), a behavioral coding system consisting of observationally-defined nonverbal signs of pain and discomfort derived from the Non-Communicating Children's Pain Checklist–Revised (34). Behaviors were scored in four categories: upper face behaviors included brow furrows, eyebrow raises, eye squeezes, narrowing of eyes, lip puckering, and rapid blinks; lower face behaviors included parted lips, mouth opening, mouth stretches, smiles, or grimaces, tongue thrusts, teeth grinding, and biting lips; body codes included flinches, movements away from the examiner, and guarding of the limb being touched (e.g., blocking the examiner's attempt with another body part); vocalizations included any vocalizations if the participant was nonverbal (e.g., moaning, grunting, yelling) and only words related to the experience if participants had verbal language (e.g., “that hurts”).

To start, coders identified behaviors that would meet the operational definitions for the codes described above but occurred repeatedly or constantly outside the context of the exam. These codes were only scored during the exam if they increased in intensity or frequency during the PEP, such as a slightly open mouth at baseline opening widely during an arm movement. This process was included to minimize the impact of movement disorders or other idiosyncratic non-pain movements on scores. Subsequently, each behavioral category was scored for each limb of the four limbs on a 0–3 scale, with 0 being no observed behaviors from that category and three being three or more seconds or three or more occurrences of a defined behavior in that category. Scores for each category were summed for a total score for each limb from 0 to 12, and a total test score of 0 to 48.

All coders were trained to a 90% or higher interobserver agreement criteria with the lead coding trainer. All videos were first coded independently by two coders, then disagreements between the two coders' score sheets were resolved *via* consensus to create a final score for each PEP. Pre-consensus IOA for this sample was 86.50% (SD = 6.13).

### Statistical Analyses

Descriptive statistics, including means and standard deviations, were calculated for all variables. Paired *t*-tests were calculated to evaluate simple change from before surgery to follow-up for each outcome. For descriptive purposes, change scores for each outcome were calculated by subtracting the value for each participant at follow-up from their value prior to surgery. Bivariate correlations were calculated for all continuous predictors and outcomes, including change scores.

Restricted maximum likelihood linear mixed models were used to evaluate change relationships between demographic factors, communication abilities, and PCS-P scores on change in pain interference, and behavioral reactivity scores. All analyses were conducted in R (35) using the lme4 (36), lmerTest (37), ggeffects (38), and cAIC4 (39) packages. *P*-values for fixed effects were estimated *via* the Satterthwaite approximation (37) for descriptive purposes only. For each outcome, a full model was calculated and then backwards elimination was used to remove uninformative terms from the models by comparing AIC values at each step (40). The model with the smallest conditional AIC value was selected as the final model (39). All models included random intercepts for participant dyads. The initial full models included time (i.e., before surgery and at follow-up), child sex, child age, GMFCS level (i.e., Levels II/III and Level V each compared to Level IV), spoken language ability, PCS-P scores, and the interaction between communication score and PCS-P score as predictors. Because preliminary analyses suggested that the “no spoken language” and “some words” groups did not differ from each other in any of the models, spoken language ability was dichotomized into “phrase speech” or “no phrase speech” for the purposes of the analyses. Each predictor was also included as an interaction with time to evaluate its effect on the slope of change. All continuous predictors were mean-centered and scaled to range from −1 to 1 to maximize the likelihood of model convergence. Time to follow-up (in weeks) was not correlated with any of the predictors or outcomes and so was not included in any models. For interpretation of reduced model results, estimated marginal means were calculated at the 25^th^ and 75^th^ percentiles from the current sample for continuous predictors as high and low values.

Although pain interference scores showed significant skewing, residuals plots from the final model showed that the assumptions of heteroscedasticity and normality of the residuals were reasonably met when using Gaussian (identity) link models for both variables. Behavioral reactivity scores were approximately normally distributed.

## Results

No significant differences were found by spoken language ability for child age or sex, parental educational attainment, race, or PCS-P, or pain interference scores. Although behavioral reactivity scores did not differ between dyads with and without phrase speech prior to surgery (*t*_27_ = 0.537, *p* =0.596), the two groups did show significant differences at follow-up (*t*_27_ = −3.992, *p* < 0.001). There was a strong association between verbal ability and reported degree of cognitive impairment (gamma = −0.763, *p* < 0.001), and between communication ability and GMFCS (gamma = −0.701, *p* < 0.001). Descriptive statistics by spoken language ability are reported in Table 2.

**Table 2:** Summary of demographic and clinical characteristics by spoken language status.

**Demographic and clinical variables**	**No phrase speech(*N* = 30)**	**Phrase speech(*N* = 10)**
***Categorical variables***	***n*** **(%)**	***n*** **(%)**
**Child sex**		
Male	18 (60)	6 (60)
Female	12 (40)	4 (40)
**GMFCS level**		
II or III	3 (10)	5 (50)
IV	6 (20)	3 (30)
V	21 (70)	2 (20)
**Intellectual disability**		
None	4 (13)	2 (20)
Mild/moderate	7 (23)	8 (80)
Severe/Profound	19 (63)	0 (0)
Parent college degree	23 (77)	6 (60)
Race = White, not Hispanic/Latinx	24 (80)	8 (80)
***Continuous variables***	***Mean (SD)***	***Mean (SD)***
Child age (months)	136.47 (57.34)	117.50 (39.51)
PCS-P scores	38.83 (11.44)	38.12 (12.00)
**Behavioral react. scores**		
Before surgery	23.65 (6.81)	21.83 (9.54)
After surgery	17.43 (7.98)	24.67 (1.75)
**Pain interference scores**		
Before surgery	45.80 (32.61)	29.30 (30.97)
Follow-up	21.77 (19.85)	23.60 (34.70)

Correlations among the pain outcomes and child age are reported in Table 3. At the bivariate level, PCS-P scores were most strongly correlated with pain interference scores before surgery (*r* = 0.422, *p* = 0.007), and at follow-up (*r* = 0.374, *p* = 0.017). Behavioral reactivity scores were not associated with parental pain catastrophizing. None of the variables were associated with child age.

**Table 3:** Bivariate relationships among all predictors and outcomes.

**Variable**		**1**	**2**	**3**	**4**	**5**	**6**	**7**
1	Pain interference T1	-						
2	Pain interference T2	0.443 (0.004)	-					
3	Pain interference change (T1-T2)	0.717 (<0.001)	−0.307 (0.054)	-				
4	Behavioral reactivity T1	−0.368 (0.049)	0.181 (0.346)	−0.522 (0.004)	-			
5	Behavioral reactivity T2	−0.237 (0.216)	0.000 (0.999)	−0.243 (0.204)	0.295 (0.120)	-		
6	Behavior reactivity change (T1-T2)	−0.096 (0.619)	0.149 (0.442)	−0.217 (0.259)	0.563 (0.001)	−0.623 (<0.001)	-	
7	Parental pain catastrophizing	0.422 (0.007)	0.374 (0.017)	0.157 (0.335)	−0.070 (0.720)	−0.031 (0.871)	−0.030 (0.878)	-
8	Child age	−0.018 (0.912)	−0.055 (0.735)	0.024 (0.884)	−0.246 (0.199)	−0.086 (0.659)	−0.127 (0.512)	−0.075 (0.647)

**Figure 1:**
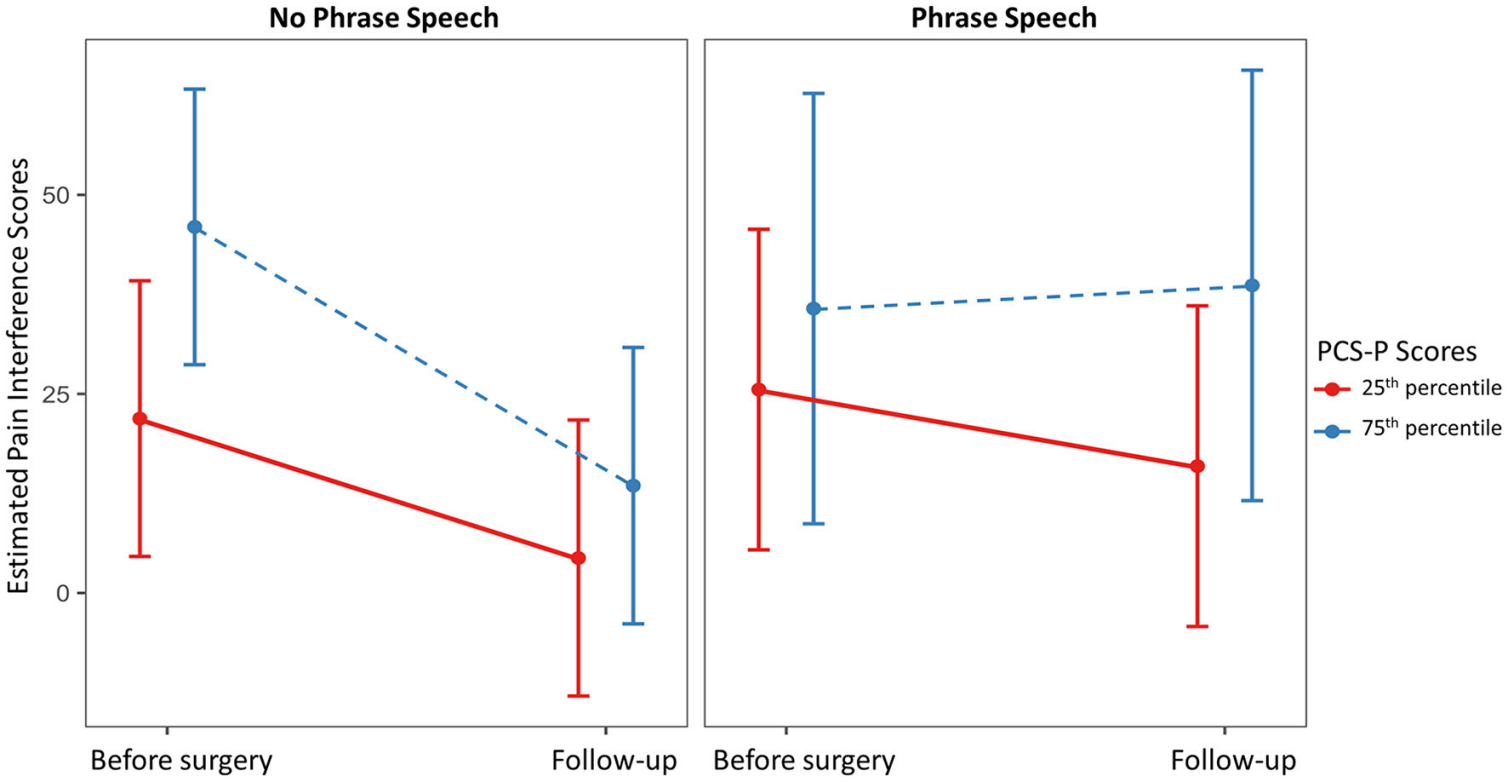
Estimated marginal mean pain interference scores at the 25^th^ and 75^th^ percentile scores on the Pain Catastrophizing Scale for Parents (PCS-P) by child spoken language ability prior to surgery and at follow-up.

Full and reduced model results for pain interference scores are presented in Table 4, and estimated marginal means are presented in Figure 1. On average, pain interference score decreased from 41.68 (SD = 32.63) prior to surgery to 22.23 (SD = 23.91) at follow-up [*t*_(39)_ = 4.00, *p* < 0.001, Cohen's d = 30.73]. Child age and sex were not maintained in the final model as main effects or interactions with time. GMFCS level was maintained as a main effect only. On average, individuals functioning at Level V (i.e., requires support to sit) had higher reported pain interference scores across both time points. The three-way interaction between spoken language, PCS-P scores, and time was retained in the reduced model. Among dyads in which the child did not use phrase speech, estimated pain interference scores estimated at the 25^th^ percentile of PCS-P scores decreased were 21.89 [95% CI: (4.58, 39.20)] prior to surgery and 4.29 [95% CI: (−12.95, 21.72)] at follow-up; at the 75^th^ percentile of PCS-P scores, estimated pain interference scores were 45.98 [95% CI: (28.68, 63.27)] and 13.48 (−3.87, 30.84). For dyads in which the child had phrase speech, at the 25^th^ percentile of PCS-P scores, estimated pain interference scores were 25.54 [95% CI: (5.41, 45.67)] and 15.93 [95% CI: (−4.20, 36.06)]; at the 75^th^ percentile of PCS-P scores, estimated pain interference scores were 35.71 [95% CI: (8.69, 62.74)] and 38.63 [95% CI: (11.60, 65.66)].

**Table 4:** Full and reduced linear mixed model results for pain interference scores.

	**Full model**					**Reduced model**				
**Model terms**	**Est**.	**SE**	**df**	***t***	***p***	**Est**.	**SE**	**df**	***t***	***p***
**Main effects**										
Time	−27.34	11.73	31.98	−2.33	0.026	−23.96	5.27	36.19	−4.55	<0.001
Female	−10.86	8.61	57.58	−1.26	0.212					
Age	−1.27	9.66	57.56	−0.13	0.896					
Phrase speech	−3.76	11.25	58.93	−0.33	0.739	−2.34	10.36	57.91	−0.23	0.822
PCS-P	99.39	28.60	57.88	3.48	0.001	97.90	27.19	60.65	3.60	0.001
GMFCS										
Level II or III	−2.67	14.30	59.86	−0.19	0.852	−6.11	11.50	38.16	−0.53	0.598
Level V	18.85	10.99	57.95	1.72	0.092	18.19	8.70	34.79	2.09	0.044
PCS-P*Phrase speech	15.26	18.28	36.18	0.84	0.409	−56.55	48.55	59.99	−1.17	0.249
**Interactions with Time**										
Sex	10.92	9.90	32.02	1.10	0.278					
Age	−0.12	11.11	31.85	−0.01	0.991					
Phrase speech	21.36	13.32	32.54	1.60	0.119	19.75	10.58	36.03	1.87	0.070
PCS-P	−62.69	32.71	32.11	−1.92	0.064	−60.92	30.38	36.34	−2.01	0.052
GMFCS										
Level II or III	−4.37	17.11	32.90	−0.26	0.800					
Level V	−0.70	12.66	31.99	−0.06	0.957					
PCS-P*Phrase speech	121.49	59.36	31.95	2.05	0.049	111.86	53.92	36.09	2.08	0.045
cAIC = 751.82						cAIC = 741.24				
df = 34.67						df = 30.05				
Conditional log-likelihood = −341.24						Conditional log-likelihood = −340.58				

**Figure 2:**
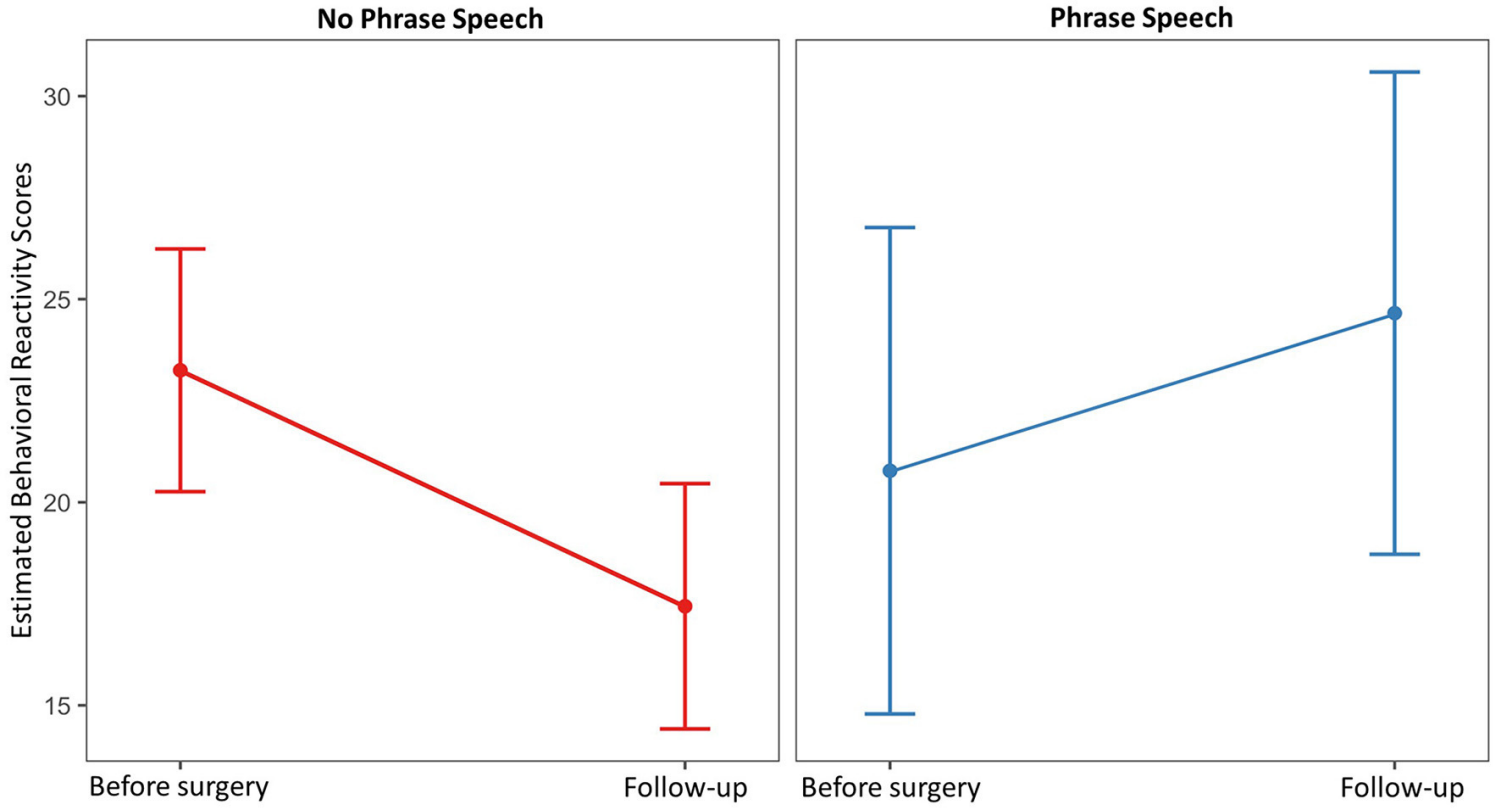
Estimated marginal mean behavioral reactivity scores prior to surgery and at follow-up by child spoken language ability.

Full and reduced model results for total behavioral reactivity are presented in Table 5. For the total behavioral reactivity during the PEP, scores decreased from an average of 23.28 (SD = 7.30) prior to surgery to 18.93 (SD = 7.71) at follow-up [*t*_(28)_ = 2.94, *p* = 0.014, Cohen's d = 8.92]. Child sex, gross motor function, and PCS-P scores were not retained in the reduced model. Only child age and phrase speech were maintained as main effects and interactions with time. Estimated marginal means over time for dyads in which the child did and did not use phrase speech are presented in Figure 2. Overall, dyads in which the child did not use phrase speech showed a decrease in behavioral reactivity from 23.25 [95% CI: (20.26, 26.24)] to 17.44 [95% CI: (14.42, 20.46)], whereas dyads in which the child used phrase speech showed no substantial change in behavioral reactivity, with a mean of 20.78 [95% CI: (14.79, 26.76)] prior to surgery, and 24.66 [95% CI: (18.72, 30.59)] at follow-up.

**Table 5:** Full and reduced linear mixed model results for behavioral reactivity scores.

	**Full model**					**Reduced model**				
**Model terms**	**Est**.	**SE**	**df**	***t***	***p***	**Est**.	**SE**	**df**	***t***	***p***
**Main effects**										
Time	−9.63	4.96	20.79	−1.94	0.066	−5.83	1.69	26.34	−3.44	0.002
Sex	−0.08	3.18	36.13	−0.02	0.981					
Age	−7.00	4.20	36.09	−1.67	0.104	−5.30	3.37	45.07	−1.57	0.123
Phrase speech	−3.78	4.10	36.49	−0.92	0.362	−2.48	3.43	45.04	−0.72	0.474
PCS-P	−12.17	9.09	36.53	−1.34	0.189					
GMFCS										
Level II or III	−0.51	5.44	37.98	−0.09	0.926					
Level V	−2.80	4.55	36.39	−0.62	0.541					
PCS-P*Phrase speech	15.26	18.28	36.18	0.84	0.409					
**Interactions with time**										
Sex	0.86	3.45	20.95	0.25	0.806					
Age	7.71	4.55	20.73	1.70	0.105	5.23	3.71	26.01	1.41	0.170
Phrase speech	11.27	4.52	20.91	2.49	0.021	9.69	3.78	26.00	2.56	0.017
PCS-P	12.31	9.81	20.99	1.26	0.223					
GMFCS										
Level II or III	2.32	6.14	21.28	0.38	0.709					
Level V	4.03	4.96	20.79	0.81	0.426					
PCS-P*Phrase speech	−14.78	19.84	20.76	−0.75	0.465					
cAIC = 406.50						cAIC = 391.53				
df = 30.53						df = 22.98				
Conditional log-likelihood = −172.72						Conditional log-likelihood = −172.78				

## Discussion

The purpose of this exploratory study was to evaluate whether child spoken language ability and parental pain catastrophizing influenced parent-reported pain and child behavioral expression of pain during a standardized pain exam among children and adolescents with CP undergoing ITB implantation. Overall, parents' pain interference ratings showed substantial decreases following ITB pump implantation, but the changes in behavioral expression of pain were modest. The interaction between PCS-P scores and child language ability scores predicted change in pain interference scores over time, with dyads in which the child used phrase speech and the parents reported high PCS-P scores estimated to have the smallest decreases in pain interference scores. As anticipated, however, higher PCS-P scores were associated with higher pain interference scores across levels of communication ability. These results suggest that, although parental negative pain cognitions appear to be related to estimated pain interference prior to surgery, relatively independent of the child's ability to communicate verbally, the change in parents' perceptions of pain interference following surgery may depend, at least in part, on the child's verbal communication ability. Nevertheless, these effects were fairly modest, and confidence intervals for the estimated pain interference scores were wide, particularly in the group with phrase speech. Further, the findings do not provide information regarding the causality of the relationship between pain interference and PCS-P scores. In the context of chronic pain, this relationship is likely bidirectional, with higher child pain leading to greater parental catastrophizing, which in turn, leads parents to restrict activities or engage in other protective behaviors that ultimately increase child disability and chronic pain (9). Still, these results provide preliminary evidence that parent pain-related cognitions and resulting behaviors that are believed to mediate the relationships between cognitions and child pain outcomes may be most pronounced among dyads in which the child has more spoken language abilities. This relationship may be mediated by differences in the child's ability to describe their pain and advocate for pain relief, how parents talk with their children about their pain, and the degree to which the children are able to imitate and learn from their parents' pain-related behaviors. Prospective longitudinal studies that evaluate parent psychosocial factors prior to the onset of child chronic pain are needed to fully understand these complex relationships.

PCS-P scores were not associated with behavioral reactivity in the current analysis. Although behavioral reactivity did not differ by spoken language ability prior to surgery, on average, individuals without phrase speech showed relatively large decreases in behavioral reactivity across time, whereas individuals with phrase speech showed no substantial change and a trend toward increasing reactivity. The reasons for this finding are unclear, although there are several potential explanations. One plausible explanation is that the finding is attributable, at least in part, to differences in cognitive ability between the two groups. Cognitive function was not formally measured in this study, and as such was not included in the statistical models. Assessing cognitive ability among children with severe communication and motor impairments is extremely challenging, but evidence suggests that communication abilities are often (but not always) correlated with cognitive ability in CP (41, 42). It is therefore plausible that individuals with more language abilities were also more aware of the context in which the study took place, and therefore more likely to exhibit anxiety in anticipation of the potentially painful exam, which may have inflated their reactivity scores at follow-up. Alternatively, it is possible that the behavioral observation scale used, which was designed to evaluate pain and discomfort among individuals with severe IDD, is not a valid measure for use among individuals without cognitive impairment. Behavioral expression of pain varies widely between individuals, and it is possible that individuals with more severe cognitive impairment may be less susceptible to social and environmental influences that may lead individuals to mask or suppress signs of pain. Scores for the two groups did not differ prior to surgery, however, suggesting that the measure captured some pain signs in this group in this context, and making this explanation less likely. Finally, because individuals with phrase speech were also more likely to have better gross motor function, it is possible that the observed differences are due to these factors as opposed to communication ability specifically. GMFCS level was included in the statistical models, however, and did not contribute to model fit for the behavioral reactivity models, suggesting that gross motor function does not account for the finding.

Although the current study was not set up to evaluate longitudinal relationships between the pain outcomes, it is notable that change in pain interference was not significantly correlated with change in behavioral reactivity. One possible explanation for these null results is that the way in which the standardized pain examination was implemented may have obscured improvements in musculoskeletal pain for some participants. Because the protocol indicated that the examiner should move each joint through the full range of motion to the degree possible, many participants likely exhibited a greater range of motion in their joints at follow-up due to the effects of the ITB pump. As a result, it is possible that changes in behavioral reactivity were not observed for some participants because there were minimal changes in the amount of pain elicited, despite likely improvements in pain during activities of daily living. Future work using similar measures should consider ways to control for such variability in the change of range of motion.

To our knowledge, this study was the first to examine the relationships among PCS-P scores, child spoken language ability, and pain outcomes among children and adolescents with IDD and associated chronic health conditions. As such, the study was exploratory in nature and all of the findings should be considered preliminary and specific to the sample. It is unclear whether these results would be replicated among samples of individuals with different etiologies of IDD, or in samples of individuals with CP without intellectual disability. Given the important role of parents as advocates for their children in healthcare contexts, future research is needed to replicate and extend these results.

The small sample size and missing data, particularly for the direct observational measure, are significant limitations of the current study. The dyads with complete data likely were not representative of the population of mother-child dyads undergoing ITB implantation. It is likely that the participants who returned to the hospital for follow-ups (as opposed to seeking follow-up care with other providers) differed from those who did not in terms of geographic location and other demographic factors. Because of the small number of father-child dyads who participated in the study, we were unable to include this group in the analyses. Preliminary analyses showed that pain catastrophizing scores, pain interference scores, and behavioral reactivity scores were all lower among father-child dyads compared to the rest of the sample, suggesting a potential influence of parent sex on parent psychosocial factors and child outcomes. Previous research has shown that mothers typically engage in higher levels of pain catastrophizing than fathers (43). As only one parent completed the measures, we were unable to consider triadic influences in two-parent families, or the role of sibling influence at the sibling subsystem or family level. Future research should consider specifically selecting for differences in family structures and expanding beyond the dyadic level to better understand these relationships.

Another limitation is that the pain measures used in the study did not specifically differentiate between acute and chronic pain. Although we attempted to isolate the effects of chronic musculoskeletal pain in the current analyses, relying on parent report of recent painful events is imperfect. Visits in which the parent reported a recent acute pain were excluded from the analyses, but this likely missed pain events that were not mentioned by or known to the parent. Nevertheless, we believe that the current results primarily reflect the impact of chronic pain in this population.

Several participating dyads did not complete the relevant questionnaire measures because translated versions of the measures were not available and the parents did not speak or read English well enough to complete them. As is the case for many pain studies, the resulting study sample was predominantly white and well-educated. Because pain experience and expression are likely influenced by family ethnocultures and microcultures (44), research in more diverse samples is needed to evaluate whether the impact of parent psychosocial factors on child outcomes varies across ethnic, racial, and socioeconomic groups.

In addition to the limitations already noted, several additional issues should be considered. Although the PCS-P has been used extensively in other populations, there are many other psychosocial variables that likely play a role in how parents rate and respond to their child's pain. For example, parent behavioral responses to pain, parent and child symptoms of anxiety and depression, and specific coping strategies have all been associated with outcomes for typically-developing children and adolescents with chronic pain [e.g., (12, 45–48)]. There is also some disagreement in the literature regarding the validity of the construct of pain catastrophizing as measured through self-report (49). Nevertheless, it remains among the most predictive psychosocial variables examined to date. Categorization of child spoken language ability was also limited; as this study was a secondary data analysis, however, no other standardized communication, cognition, or adaptive behavior measures were collected. Because the phrase speech variable was based primarily on the behavior of the individual with CP during study visits, it is possible that some individuals with the ability to use phrase speech were missed. The measure also did not take into consideration receptive language ability or nonverbal forms of communication, including formal augmentative and alternative communication systems and devices.

Many different measures exist for the assessment of pain and discomfort among individuals with CP and IDD, and selection of measures likely influences their relationships with parental variables. The pain interference scale was selected for the current analysis because it is a global measure of the parents' perceptions of the degree to which their child's quality of life is impacted by pain, as opposed to a more direct measure of pain intensity. We hypothesized that this would be more closely related to parents' levels of pain catastrophizing than other parent-reported pain measures, although there is no work investigating these relationships. The direct observation measure was selected because it was specifically designed to elicit signs of the musculoskeletal pain that was considered to be most relevant in the current context. Although both measures used in the current study have previously been documented as useful pain assessments among children with CP, there is no consensus regarding the most appropriate measures for this population, and development and validation is an ongoing process. Future research is needed to evaluate the relationships between parent psychosocial factors and the various pain assessment methods and to determine the most useful measures for specific purposes.

Despite these limitations, the results of the current study provide preliminary evidence that parent and child factors may influence proxy report measures of pain in children and adolescents with CP, although child factors appear to be more relevant for the direct observational measure. Palermo and Chambers suggest an integrative framework for the role of parent and family factors in a child's pain (2005). In this framework, pain expression in the family is a reciprocal process influenced by a child's developmental status that occurs within an ecological context (9). Parent catastrophizing or child verbal ability could be understood as individual variables that impact the dyadic and family levels. At the dyadic level, the parent's catastrophizing influences the child's catastrophizing and the child's verbal ability impacts the parent's perceptions of the child's pain interference (19). For example, the child's pain expression may be influenced by their parent's modeling of pain expression over time, including facial expressions, vocalizations, and gestures. The parent's perception of pain interference may be influenced by the child's verbal complaints or requests for analgesics, comfort, or rest.

At the family level, the family microculture around pain expression and management may be influenced by pain catastrophizing and/or child verbal ability. Consistent with the communal coping model of pain, Kraljevic et al. describe a family's microculture around pain as a “specific cognitive style for coping with pain, which is associated with a child's responses to pain experiences” (2011, p. 115). Over the long term, catastrophizing may adversely affect family atmosphere; this is likely bidirectional, as there is also evidence that family dysfunction predicts catastrophic thinking (50). When a child has significant disabilities associated with chronic pain, such as cerebral palsy, the risk for adverse effect on family atmosphere likely becomes more pronounced. Child verbal ability is also likely to have an influence on the family microculture as it relates to their pain; the family's communal coping style and pain management patterns may more or less depend on verbal cues.

There is an urgent need to understand the variables that may influence proxy report of pain for individuals with IDD because of the clinical implications of analgesic decision-making in this vulnerable population. This is the first investigation showing that parent-reported pain interference and behavioral reactivity in the context of a standardized pain exam vary according to both parent (pain catastrophizing) and child (communication ability) psychosocial factors. The construct of pain catastrophizing and how it relates to parents serving as proxy pain reporters for their child with a developmental disability needs further investigation, as does the construct of child communication ability, and this investigation should be considered in the context of parent-child dyadic and family level interactions over time.

## Data Availability Statement

The raw data supporting the conclusions of this article will be made available by the authors, without undue reservation.

## Ethics Statement

The studies involving human participants were reviewed and approved by University of Minnesota Medical IRB. Written informed consent to participate in this study was provided by the participants' legal guardian/next of kin.

## Author Contributions

BB was substantially responsible for data analysis and interpretation of the results, drafting, and revision of the manuscript. CR was substantially responsible for the initial draft of the manuscript, supporting interpretation of the results, and revision of the manuscript. CB was substantially involved in conceptualizing the study, collecting the data, and revision of the manuscript. AM was substantially involved in data collection and analysis, and revision of the manuscript. KC was involved in the initial conceptualization of the study, and in revision of the manuscript. FS was substantially responsible for initial conceptualization of the study, data collection, and revision of the manuscript. All authors contributed to the article and approved the submitted version.

## Funding

The work was supported, in part, by Eunice Kennedy Shriver NICHD Grant No. 73126.

## Conflict of Interest

The authors declare that the research was conducted in the absence of any commercial or financial relationships that could be construed as a potential conflict of interest.

## Publisher's Note

All claims expressed in this article are solely those of the authors and do not necessarily represent those of their affiliated organizations, or those of the publisher, the editors and the reviewers. Any product that may be evaluated in this article, or claim that may be made by its manufacturer, is not guaranteed or endorsed by the publisher.
